# Complete genome analysis of the African swine fever virus isolated from a wild boar responsible for the first viral outbreak in Korea, 2019

**DOI:** 10.3389/fvets.2022.1080397

**Published:** 2023-01-11

**Authors:** Garam Kim, Jung-Eun Park, So-Jeong Kim, Yeonji Kim, Wonjun Kim, Yong-Kwan Kim, WeonHwa Jheong

**Affiliations:** Wildlife Disease Response Team, National Institute of Wildlife Disease Control and Prevention (NIWDC), Gwangju, Republic of Korea

**Keywords:** African swine fever, African swine fever virus, central variable region (CVR), complete genome, multigene families, phylogenetic analysis

## Abstract

African swine fever (ASF), a highly contagious and severe hemorrhagic viral disease in swine, is emerging as a major threat not only in Korea but also worldwide. The first confirmed case of ASF in Korea was reported in 2019. Despite the occurrence of ASF in Korea, only a few studies have genetically characterized the causative ASF virus (ASFV). In this study, we aimed to genetically characterize the ASFV responsible for the 2019 outbreak in Korea. The genome of the ASFV isolated during the first outbreak in Korea was analyzed. The Korea/YC1/2019 strain has 188,950 base pairs, with a GC content of 38.4%. The complete genome sequence was compared with other ASFV genomes annotated in the NCBI database. The Korea/YC1/2019 strain shared the highest similarity with Georgia 2007, Belgium 2018/1, and ASFV-wbBS01 strains. This study expands our knowledge of the genetic diversity of ASFV, providing valuable information for epidemiology, diagnostics, therapies, and vaccine development.

## 1. Introduction

African swine fever (ASF) is caused by the African swine fever virus (ASFV) in pigs and wild boars; it is highly contagiousness and associated with high mortality (1). ASF is mainly transmitted through direct contact between infected and susceptible domestic pigs, and through indirect contact with contaminated pork, vehicles, and excrement and infected humans (2, 3). In 2007, ASFV was introduced to Georgia through the port of Poti as a potential contaminant in food used as swine feed (4). It has since spread rapidly to many countries including China, Vietnam, Cambodia, Hong Kong, North Korea, Laos, Myanmar, the Philippines, and South Korea (5, 6). The first case of ASF in Korea occurred in September 2019 at a pig farm in Paju, Gyeonggi-do (7). In the wild, ASF was first reported on Yeoncheon in October 2019 in wild boar (8). In 2019, after the outbreak, it was limited to Yeoncheon, Cheolwon, and Hwacheon, but since 2020, ASF has spread to the east and south (9). During an ASF outbreak, analysis of the ASFV genome has proven to be the most useful tool for tracing the origin of ASFV (10). The full-length sequence of pig farm generated from farms in Paju (MT748042) and Yeoncheon (MW049116) in 2019 is registered with NCBI. The partial sequencing of the wild boar occurrence has been partially performed, but the genome information has not been revealed yet.

ASFV is a large double-stranded DNA virus belonging to the family *Asfaviridae* (11). The ASFV genome is 190–193 kb in size and encodes more than 150 open reading frames, with the central regions of the genes being highly conserved (12, 13). Although the biological functions of ASFV genes include nucleotide replication, messenger RNA processing, structural protein synthesis, and host defenses modulation, the functions of more than half of the genes in the ASFV genome are still unknown (12, 14). As the ASFV genome is large and complex, it is difficult to develop vaccines and drugs (15).

A comparative analysis of molecular properties of specific regions of the ASFV genome has proven useful in elucidating the origin and transmission pathways of ASFV during ASF outbreaks (16). Based on the p72 major capsid protein-encoding gene of ASFV *(B646L)*, the virus can be classified into at least 24 ASFV genotypes (16). The central variable region (CVR) within the *B602L* gene has been shown to be informative about the relationship between isolates at the genotype, national, and regional levels (17). A recent study revealed that a tandem repeat sequence (TRS) located in the intergenic region between *I73R* and *I329L* can be used to determine the origin of ASFV isolates (18). Additionally, the analysis of other sequences, such as *EP402R* and *E183L*, may help improve molecular epidemiological studies of ASFV (19, 20). To better understand and control the spread of ASF, it is necessary to analyze the genetic characteristics of ASFV strains. However, in South Korea, there have been no studies to characterize the complete genome of the ASFV strain responsible for the occurrence of ASF. In this study, we analyzed the complete genome sequence of the ASFV strain, which first occurred in Korea in 2019, and compared it with strains from neighboring countries.

## 2. Materials and methods

### 2.1. Analysis of distribution of ASF outbreaks

**Figure 1:**
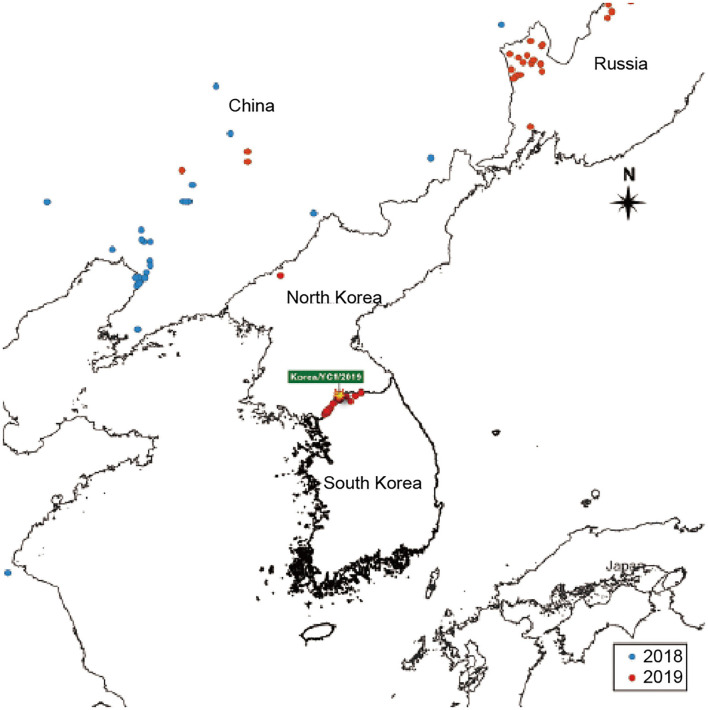
ASF-affected areas are represented. South Korea is indicated on the map. The outbreaks in 2018 and 2019 are indicated with blue and red dots, respectively.

Data for this study were acquired from the Pig Progress website (http://www.pigprogress.net), and they are presented in Figure 1. The data pertaining to ASF outbreaks re-ported in China, Russia, and North Korea from 2018 to 2019 were retrieved from this database. A cartographical analysis of the geological location of ASF outbreaks was performed using the open-source Geographic Information System (GIS) software.

### 2.2. Detection of ASFV in wild boar samples

DNA was extracted from the blood of a wild boar using the Maxwell RSC Viral Total Nucleic Purification Kit (Promega, Madison, WI, USA) following the manufacturer's instructions. The presence of ASFV DNA was detected via polymerase chain reaction (PCR) using the ASFV diagnostic primers *PPA1* (5**′**-AGTTATGGGAAACCCGACCC-3**′**), *PPA2* (5**′**-CCCTGAATCGGAGCATCCT-3**′**) (21), P72D (5**′**-GTACTGTAACGCAGCACAG-3**′**), and *P72U* (5**′**-GGCACAAGTTCGGACATGT-3**′**) (16), which partially amplified *B646L* (*p72*).

### 2.3. Genetic characterization of ASFV and phylogenetic analysis of *B646L* (*p72*)

To demonstrate the phylogenetic organization of the ASFV, we selected 16 related viruses from the initial phylogeny and built a maximum-likelihood phylogenetic tree of the whole genome sequences with RAxML version 8.0.0 (REF) using default parameters and a general time-reversible model with gamma-distributed rate variation among sites. The entire genome sequences were aligned using the MAFFT in Geneious Prime software. To assess relatedness support, maximum likelihood (ML) phylogenetic tree of the p72 gene was constructed under the MEGA. ML bootstrapping was performed with 1,000 replicates to assess the robustness of tree topologies.

The nucleotide sequence of *B646L* (*p72*) of the ASFV Korea/YC1/2019 strain was aligned with that of other ASFV strains representing the *B646L* (*p72*) genotype using the ClustalW algorithm in MEGA X. The evolutionary history was inferred using the Maximum Composite Likelihood model method. Phylogenetic analysis was performed using the neighbor-joining method with 1,000 bootstrap replications.

### 2.4. Complete genome sequencing of ASFV

The total DNA was extracted directly from 200 μl of whole blood from a wild boar using the Maxwell Viral Total Nucleic Purification Kit (Promega) following the manufacturer's recommendations. To detect the presence of ASFV, PCR amplification of the samples was performed as described in the World Organization for Animal Health (OIE) manual using primers for *PPA1/PPA2* and *P72*. gDNA was sheared and made library preparation using Enzymatic Preparation Kit (Celemics, Seoul, Republic of Korea). Prepared gDNA library and capture probes were hybridized to capture target regions through the use of Celemics target enrichment kit (Celemics, Seoul, Republic of Korea). Capture probes were designed and chemically synthesized to hybridize target region. Captured regions were then further amplified by post-PCR to enrich the amount of sample. The target-captured library were then sequenced on an Illumina NextSeq550 instrument (Illumina, San Diego, CA, USA) using the read layout 2 × 150 bp. The adaptor sequences and low quality bases were first trimmed using Fastx Toolkit (fastx_toolkit 0.0.14). The exact sequence that trimmed with the AdapterRemoval (version 2.2.2). The reads were mapped on reference ASFV genomes (accession number: FR682468) using Burrows-Wheeler Aligner software version 0.7.10. SNP, InDel, and SV variations were detected using GATK (Genome Analysis TK 4.0.4.0). The quality of read alignment was assessed using the SAMtools software (samtools 1.1) and the Python software package (numpy 1.11.0). The ASFV complete genome Korea/YC1/2019 was annotated with the genome annotation transfer utility (GATU) software using the genome of ASFV Georgia2007 as the reference.

## 3. Results

### 3.1. First outbreak of ASF in wild boars in the Korean Peninsula

To analyze the epidemic situation in Korea and neighboring countries, data on ASF outbreaks notified by the OIE from 2018 to 2019 were retrieved. The ASF outbreaks in Korea and neighboring countries from 2018 to 2019 are shown in Figure 1. The first ASF outbreak in Asia occurred in China in 2018 and subsequently spread to Mongolia, Vietnam, Cambodia, North Korea, Laos, Philippines, and Myanmar (5, 6). As shown in Figure 1, ASF was detected in a pig farm in North Korea (in Jagang-do) on May 25, 2019. Five months later, ASF occurred in South Korea. ASF spread to Yeoncheon, South Korea in September 2019. Since then, the distribution of ASF has spread rapidly. Continued outbreaks of ASF in South Korea have raised awareness regarding the negative effects of ASF on the pork industry.

### 3.2. Characteristics of the complete genome sequence of ASFV Korea/YC1/2019

**Figure 2:**
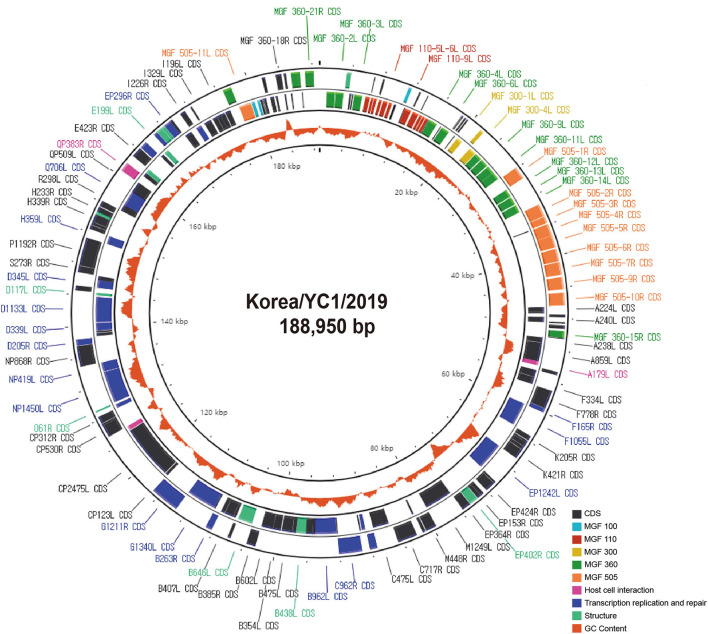
Circular genome as generated using the CGView Server. Circles show coding regions on forward strand (outer circle) and reverse strand (middle circle) and the GC content (orange inner circle).

The genome of Korea/YC1/2019 strain was successfully obtained from the blood of the first ASFV-positive wild boars in Korea, Yeoncheon, 2019 (10). In order to characterize the ASFV Korea/YC1/2019 strain, Illumina reads were aligned against the Georgia 2007/1 reference sequence (Table 1). The complete genome generated via genome assembly was 188,950 bp with a GC content of 38.4%, and 183 open reading frames were annotated using CGView (Circular Genome Viewer) (Figure 2). Coverage for the forward and reverse strands is shown in the outer and middle circles, respectively. The ORFs of the Korea/YC1/2019 strain contains those that encode 4 proteins involved in host cell interactions, 16 structural proteins, and 25 proteins involved in nucleotide metabolism, transcription, replication, and repair. Furthermore, 47 MGF members were identified within the genome of Korea/YC1/2019 strain including MGF100 (3 members), MGF110 (12 members), MGF300 (3 members), MGF360 (19 members), and MGF505 (10 members) (Figure 2).

**Table 1:** Summary of the Korea/YC1/2019 strain genomic sequencing data.

**Strain**	**Sample type**	**Raw reads**	**Filtered read**	**ASFV read (pre-filtered)**	**Mean coverage (pre-filtered)**	**ASFV read (post-filtered)**	**Mean coverage (post-filtered)**
Korea/YC1/2019	Blood	13,496,754	13,451,232	13,028,940	7,511	10,048,618	5,917

To determine genetic relationships at the whole-genome level, we performed multiple sequence alignments of the whole-genome sequences. The 29 strains are listed by accession strain name, accession number, country, and year of isolation in Table 2. The newly determined Korea/YC1/2019 genome with 188,950 bp is shorter than Georgia 2007 (190,584 bp) and Belgium 2018/1 (190,599 bp) genomes (Table 2). The complete genome of Korea/YC1/2019 exhibited 99.9% nucleotide identity with Georgia 2007/1, China/CAS19-01/2019, HLJ/2018, POL/2015/Podlaskie, and China/2018/AnhuiXCGQ genomes (Table 2). The nucleotide identity with other ASFV p72 genotype II derived from wild boars, such as Belgium 2018/1 and ASFV-wbBS01, was 100% (Table 2). In contrast, the Estonia 2004 strain (genotype II) exhibited 97.8% nucleotide identity with the Korea/YC1/2019 strain (Table 2). Unlike ASFV genotype II viruses, including the Korea/YC1/2019 strain, ASFV Estonia 2004 exhibited deletion and specific rearrangement at the 5′ end, which resulted in reduced virulence (26).

**Table 2:** Comparison of genome features of different ASFV strains and Korea/YC1/2019.

**Strain**	**Accession number**	**Country**	**Year**	**Length (bp)**	**Genotype**	**Identity to Korea/YC1/** **2019**	**References**
Georgia 2007/1	FR682468.2	Georgia	2007	190584	II	99.9	(22)
Arm/07/CBM/c2	LR812933	Armenia	2007	190145	II	99.9	(23)
Tanzania/Rukwa/2017/1	LR813622	Tanzania	2007	183186	II	99.9	(24)
Odintsovo_02/14	KP843857	Russia	2014	189333	II	99.9	(25)
Estonia 2014	LS478113	Estonia	2014	182446	II	97.8	(26)
POL/2015/Podlaskie	MH681419	Poland	2015	189394	II	99.9	(27)
Belgium 2018/1	LR536725	Belgium	2018	189404	II	99.9	(28)
ASFV-wbBS01	MK645909	China	2018	189394	II	100	Unpublished
China/2018/AnhuiXCGQ	MK128995	China	2018	189393	II	99.9	(29)
ASFV_HU_2018	MN715134	China	2018	190601	II	99.9	(30)
Pig/HLJ/2018	MK333180	China	2018	189404	II	99.9	(19)
CAS19-01/2019	MN172368	China	2019	189405	II	99.9	(31)
Wuhan2019-1	MN393476	China	2019	190576	II	99.9	Unpublished
Ulyanovsk 19/WB-5699	MW306192	Russia	2019	189263	II	99.9	(32)
Portugal/L60	KM262844	Portugal	1960	182362	I	92.7	(33)
BA71	KP055815	Spain	1971	180365	I	90.9	(34)
Spain/E75	FN557520	Spain	1975	181187	I	92.6	(35)
Mkuzi1979	AY261362	South Africa	1979	192714	I	95.7	Unpublished
Benin 97/1	NC_044956	Benin	1997	182284	I	92.7	(11)
47/Ss/2008	KX354450	Italy	2008	184638	I	92.4	Unpublished
Warmbaths	AY261365	South Africa	1987	190773	III	94.5	Unpublished
Namibia/Warthog	AY261366	Namibia	1980	186528	IV	93.1	(36)
Ken06.Bus	KM111295	Kenya	2006	184368	IX	84.7	(37)
Uganda/R35	MH025920	Uganda	2015	188629	IX	86.8	Unpublished
Tengani62	AY261364	Malawi	1962	185689	V	91.9	Unpublished
MalawiLil-20/1	AY261361	Malawi	1983	187162	VIII	87.7	(38)
Kenya1950	AY261360	Kenya	1950	193886	X	86.1	(37)
BUR/18/Rutana	MW856067	Burundi	2018	176564	X	83.5	(39)
ASFV Ken.rie1	LR899131	Kenya	2019	190592	X	86.8	Unpublished

**Figure 3:**
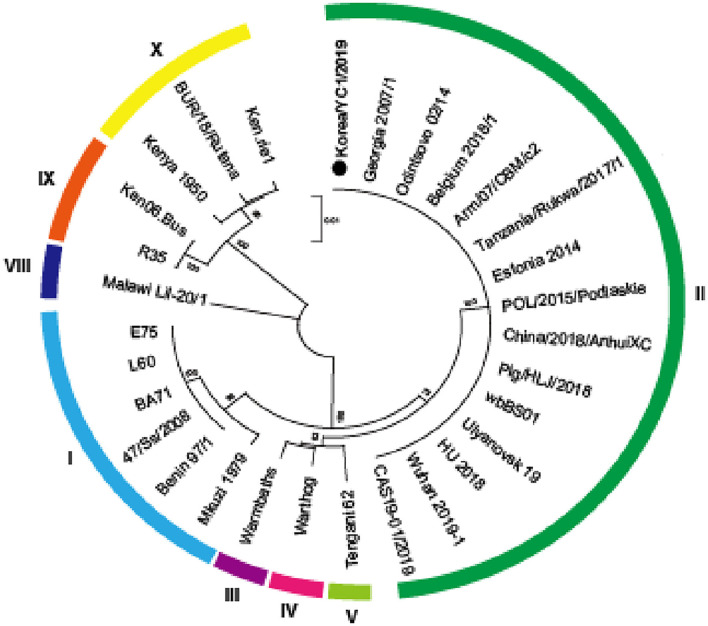
Phylogenetic relationship of ASFV strains based on *p72* (*B646L*). A phylogenetic tree based on the full-length *p72* sequence alignment of the Korea/YC1/2019 strain and 29 public ASFV strains. ASFV Korea/YC1/2019 isolated in this study is marked with a black dot. ASFV strains are named by their isolate names. Genotypes I, II, III, IV, V, VIII, IX, and X are labeled in sky blue, green, purple, pink, yellow-green, blue, yellow, and orange, respectively. Phylogeny was inferred following 1,000 bootstrap replications and node values show percentage bootstrap support. The scale bar indicates nucleotide substitutions per site.

In previous studies, the Korea/YC1/2019 strain was identified as a member of the genotype II group on the basis of *B646L* encoding the capsid protein p72 (10). The partial nucleotide sequence of *B646L* of the Korea/YC1/2019 strain was aligned with the sequence of the indicated ASFV strains using the ClustalW algorithm in MEGA X. The phylogenetic analysis based on the *B646L* gene sequences revealed that the 29 strains were grouped into eight genotypes (Figure 3). A comparison of ASFV sequences revealed 100% similarity between the Korea/YC1/2019 strain and the Georgia 2007 and China/CAS19-01/2019 strains.

Several studies have demonstrated that tandem repeat sequences (TRS) located in the CVR of *B602L* are suitable as genetic markers for distinguishing the genotype of ASFVs (16). The CVR tetrameric repeats of ASFV in the Korea/YC1/2019 strain included CADT, NVDT, CASM, CAST, and CSTS, which correspond to the B, N, D, and A codes, respectively. The results showed a single TRS profile of 10 amino acid tetramers “BNDBNDBNAA” in Korea/YC1/2019, with 100% sequence identity to the Georgia 2007 strain (Table 3).

**Table 3:** Tetrameric amino acid sequence in the central variable region (CVR) of *B602L* in ASFV strains.

**Strain**	**CVR (aa sequence)**	**No. of repeats**	**IGR type**
Korea/YC1/2019	BNDBNDBNAA	10	II
Georgia 2007/1	BNDBNDBNAA	10	I
China/CAS19-01/2019	BNDBNDBNAA	10	II
Belgium 2018/1	BNDBNDBNAA	10	II
ASFV-wbBS01	BNDBNDBNAA	10	I
Arm/07/CBM/c2	BNDBNDBNAA	10	I
Wuhan2019-1	BNDBNDBNAA	10	II
Tanzania/Rukwa/2017/1	BNDBNDBNAA	10	I
China/2018/AnhuiXCGQ	BNDBNDBNAA	10	II
POL/2015/Podlaskie	BNDBNDBNAA	10	I
Odintsovo_02/14	BNDBNDBNAA	10	II
Estonia 2014	BNDBNDBNAA	10	II
ASFV_HU_2018	BNDBNDBNAA	10	I
Ulyanovsk 19/WB-5699	BNDBNDBNAA	10	III
Pig/HLJ/2018	BNDBNDBNAA	10	III
Benin 97/1	ABNAAAAFBNAAAAAFBNAAAAAFBNAAAAFBNAFA	36	I
Mkuzi1979	BVWAFNBNAAAF	12	I
Porutugal/L60	ABNAAAAFBNABNABNABNVNTDBNAFA	25	I
47/Ss/2008	ABNAAADBNAFA	12	I
Spain/E75	ABNAAAAAFBNABNABNAB	26	I
BA71	ABNAAAAFBNABNABNABNABNVNTDBNAFA	28	I
Warmbaths	BVWVWVVNAABAG	13	I
Namibia/Warthog	BNAB	4	I
Tengani62	ABNBBMA	7	III
MalawiLil-20/1	AVSVSOVNAVNOVVNVOVNAVNOVVNOVOOV	31	I

Letters in CVR sequence represent the TRS previously characterized in ASFV isolates: A = CAST, CVST, CTST, CASI; B = CADT, CADI, CTDT, CAGT, CVDT; F = CANT, CAAT; N = NVDT, NVGT, NVDI; T = DVDT, NVNT; S = SAST; O = NANI, NADI, NASI; V = NAGT, NAST, NAVT, NADT, NANT, NANV; D = CASM; G = CTNT; M = NEDT; W = SADT, SVDT.

**Figure 4:**
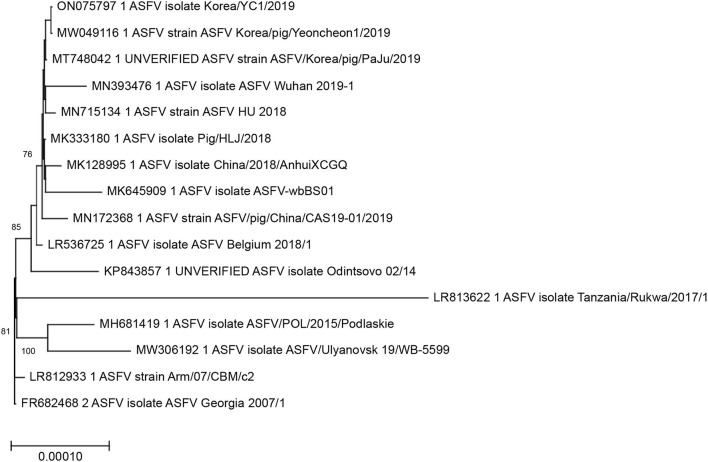
Maximum-likelihood analysis of 16 complete coding sequences of ASFV including the Korea/YC1/2019 strain sequenced in this study. The phylogeny was rooted at the Georgia/2007 virus. The scale bars show the number of substitutions per site. The numerical values represent 1,000 bootstrap replicate values >70 expressed as a percentage.

The ASFVs detected in Korea clustered together in the whole genome ML phylogeny, suggesting high genetic relatedness of the viruses. The ASFVs detected in Korea and China shared a common ancestry and formed a well-supported monophyletic cluster with high bootstrap support (>70%), showing that the ASFV isolates detected in Korea are most likely descendants of the viruses that circulated in China (Figure 4).

**Figure 5:**
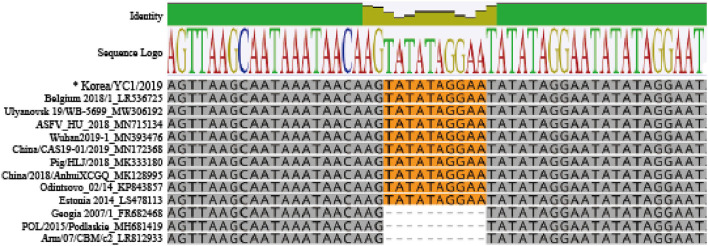
Sequence alignment of intergenic region between *I73R* and *I329L* in ASFV genotype II and Korea/YC1/2019. Orange shading on the sequences indicates the 10-nucleotide tandem repeat sequence (TRS) insertion.

Moreover, the Korea/YC1/2019 strain had numerous insertions corresponding to the 10-nucleotide sequence TATATAGGAA, a TRS between *I73R* and *I329L* (Figure 5). A previous study has reported that the insertion in this region has no relationship with attenuation or virulence of the ASFV (18). This insertion was present in the genome of Belgium 2018/1 (accession no. LR356725), but absent in the genome of POL/2015/Podlaskie (accession no. MH681419) and Georgia 2007/1. The generated complete genome sequences were submitted to GenBank and assigned an accession number (ON075797 for Korea/YC1/2019).

## 4. Discussion

ASFV partial sequencing data are commonly used to determine ASFV genotypes and distinguish related ASFV strains. However, studies on complete genome sequencing are required to obtain adequate information about ASFV genetic variation and molecular evolution events. In this study, we characterized the genome of the ASFV strain that caused the first outbreak of ASF in Korea in 2019. A comparison of the genome sequence of the ASFV Korea/YC1/2019 strain with the sequences of other p72 genotype II strains showed that it shared similarities with Georgia and China strains. As a result of their recent introduction to Georgia and the subsequent rapid dissemination north of the Russian Federation and east to China and Southeast Asia, the genotype II p72 group ASFV represents by far the most geographically widespread of the 24 viral genotypes (16, 22). The virus responsible for the 2019 ASF outbreak in Korea clustered into p72 genotype II and showed high nucleotide identity with ASFV strains causing outbreaks in neighboring countries, suggesting that the same ASFV strains are causing outbreaks across borders. Moreover, recent molecular epidemiological analyses have indicated that only genotype II appears to be widespread in Korea. Molecular characterization of the ASFV Korea/YC1/2019 strain showed that it is highly homologous and almost identical to the samples that were obtained from Georgia (2007), Russia (2012), Estonia (2014), and China (2018) (40). The onset of epidemics in different countries with similar characteristics, despite very different dates of initiation, suggests that outbreaks originated from a single source and then propagated across Asia. The spread of ASF across Asia has been largely unidirectional.

The gene encoding p72, *B646L*, is relatively conserved. Therefore, p72 clustering is the preferred method for identifying the origin of ASFV because it can help trace the source of the virus at the molecular level, offering insights into possible transmission routes. Other genotypes can also be used, such as *E183L* (p54), *CP204L* (p30), *B602L* (CVR), and TRS (16–18, 41–43). The previous studies provide useful information for molecular characterization of ASFV strains. According to previous studies on partial gene sequencing, the B602L, CVR, and TRS genotypes were found in these ASFV strains circulating in Europe and recently in China (42). A comparison of the IGR sequences of the Korea/YC1/2019 strain and the Korean-type Paju strains Korea/19S3965/wb/2019 (MT300324) and Korea/19S5464/wb/2019 (MT300325) revealed a deletion or insertion in each Paju strain, suggesting that it may have a different origin.

The dsDNA genome (170–194 kb) of ASFV, with repeats and scattered invert-ed-terminal-repeats (ITR), hamper whole-genome sequencing (14). The total length of the ASFV Georgia 2007/1 genome is 190,594 bp, 5′-ITR region is 956 bp, 3′-ITR region is 231 bp, and total length of each ITR region is 1,378 bp, with ORF DP60R and ASFV G ACD 01990 annotated ITR regions (44). Inverted terminal repeats were missing from both ends of the Korea/YC1/2019 genome sequence, presumably because of the complexity of the ASFV material or region in the limited number of sequenced DNA samples and/or the difficulty of sequencing/assembly. In addition, the Illumina sequencing platform only generates short reads, which makes it difficult to effectively sequence and assemble repeat regions, as short repeat repeats may collapse (45).

ASF was first discovered in Korea in 2019, and its outbreaks have been occurring since then. In this study, we analyzed the genome of ASFV in wild boars in Korea in 2019 for the first time. The results of whole-genome sequencing of ASFV in this study can provide information on genetic variation and help track sources and entry points. In Korea, 50 wild boars were infected with ASFV in 2019, 814 in 2020, and 964 in 2021. From 2019 to 2021, the number of benign cases in hunting animals seems to have steadily increased to 5, 40, and 116 cases in 2019, 2020, and 2021, respectively. Accordingly, with the increasing incidence of ASF in wild boars, more studies are being conducted to trace the genetic mutations in ASFV. We plan to analyze the whole ASFV genomes by year and region to continuously track the transmission route and genetic variation.

## 5. Conclusions

Here, we report the genome characterization of the ASFV Korea/YC1/2019 strain. This whole-genome characterization of ASFV may contribute to tracing the evolution of ASFV during its spread. The genetic analysis showed that the Korea/YC1/2019 strain is closely clustered with genotype II ASFVs, providing insights into the 2019 ASF outbreak. In addition, analysis of these results will provide valuable information for the improvement of ASF diagnostic methods and vaccine development, as well as epidemiological evidence that can be used to trace the virus in a specific region. Further studies of the emerging ASFVs are needed to provide more insights into genetic characterization and variations.

## Data availability statement

The datasets presented in this study can be found in online repositories. The names of the repository/repositories and accession number(s) can be found below: https://www.ncbi.nlm.nih.gov/genbank/, ON075797.

## Author contributions

Conceptualization: WJ and GK. Formal analysis: YK. Investigation: J-EP, WK, and Y-KK. Data curation: S-JK. Writing—original draft preparation, writing—review and editing, and project administration: GK. Supervision: WJ. All authors have read and agreed to the published version of the manuscript.
